# Ethical issues in the CoLaus study concerning collaborations of academia and industry

**DOI:** 10.1186/1753-6561-7-S5-O22

**Published:** 2013-08-30

**Authors:** P Vollenweider

**Affiliations:** 1 University Hospital of Lausanne, Switzerland

## 

This presentation gave an introduction to the legal background in Switzerland and the informed consent process adopted in the CoLaus study. The information covered the scope of study, risks and benefits of participation, confidentiality of data, collaboration with academia, industry and their partners, potential shipment of the samples to other collaborators, right to withdrawal, right to have samples destroyed. There were no rights to financial benefits and genetic data for the individual participant. The limitations of the consent included the broad definition of study areas and the potential conflict with right to information in future.

Collaboration with local ethics committees is important with strict protocols. Ethics committee should be informed and updated of the constant progress of technologies and feed-back from investigators.

Collaboration with industry had the benefits of significant financial input, high standards and long experience with ethical committees , long experience with material and data transfer contracts and bio-bank and database organization. The disadvantages included lengthy contracts, complicated legal issues and the negative attitude of some subjects towards involvement of pharmaceutical companies. The experience with industrial collaboration reinforces the need for transparency in all procedures, regular conferences and a strong legal department in the academic institution. Ethical dilemmas included the public availability of individual genetic data, return of genetic data to the subject and the need of multi-layered consent. CoLaus investigators decided that in general no individual data would be made publicly available.

